# Characterization of Sheep Milk Extracellular Vesicle-miRNA by Sequencing and Comparison with Cow Milk

**DOI:** 10.3390/ani10020331

**Published:** 2020-02-20

**Authors:** Suyu Quan, Xuemei Nan, Kun Wang, Linshu Jiang, Junhu Yao, Benhai Xiong

**Affiliations:** 1 State Key Laboratory of Animal Nutrition, Institute of Animal Sciences, Chinese Academy of Agricultural Sciences, Beijing 100193, China; quansuyu@163.com (S.Q.); xuemeinan@126.com (X.N.); cang327@163.com (K.W.); 2 College of Animal Science and Technology, Northwest A&F University, Yanglin 712100, China; 3 Beijing Key Laboratory for Dairy Cow Nutrition, Beijing University of Agriculture, Beijing 102206, China; jls@bac.edu.cn

**Keywords:** sheep, cow, milk, extracellular vesicle, miRNA

## Abstract

**Simple Summary:**

Milk provides significant health benefits for mammals, especially as the primary nutrient sources of neonates. Milk was recognized as the epigenetic “doping system” during mammalian development in recent research due to the bioactive component extracellular vesicles, which can act as mediators of intercellular communication, and even as signal carriers between different individuals, especially mothers and babies, by the containing regulatory miRNAs. It is widely accepted that although cow milk is generally more consumed by humans, sheep milk is more suitable for infants. However, compared with cow milk, few studies have focused on the sheep milk extracellular vesicles, which select specific types of miRNA to encapsulate. This study clarified the sheep milk extracellular vesicle-miRNA profiling by sequencing and made a comparison with that of cow milk. The results of this study provide more documents for the milk bioactive components in different species.

**Abstract:**

Milk can mediate maternal-neonatal signal transmission by the bioactive component extracellular vesicles (EVs), which select specific types of miRNA to encapsulate. The miRNA profiling of sheep milk EVs was characterized by sequencing and compared with that of cow milk. Nanoparticle tracking analysis revealed that the concentration of sheep milk EVs was 1.3 ± 0.09 × 10^12^ particles/mL and the diameter was peaked at 131.2 ± 0.84 nm. Sheep milk EVs contained various small RNAs, including tRNA, Cis-regulatory element, rRNA, snRNA, other Rfam RNA, and miRNA, which held about 36% of all the small RNAs. In total, 84 types of miRNA were annotated with *Ovis aries* by miRBase (version 22.0) in sheep milk EVs, with 75 shared types of miRNAs in all samples. The miR-26a, miR-191, let-7f, let-7b and miR-10b were highly expressed both in cow and sheep milk EVs, and 14 sheep milk EV-miRNAs in the top 20, occupying 98% of the total expression, were immune-related. Although pathway analysis showed different potential functions of cow and sheep milk EV-miRNAs, there were still some shared points: lipid metabolism (phospholipase D, glycerophospholipid and glycosylphosphatidylinositol), calcium metabolism, and nerve conduction (axon guidance and synapse). This study provides reference for the bioactive components in the milk of different species.

## 1. Introduction

Milk provides significant health benefits for both humans and other mammals, especially as the primary nutrient sources for mammals before weaning. Milk helps to shape the intestinal microbiome and contributes to the immune maturation and organ development of infants [1]. Moreover, milk can decrease the risk of allergy, sudden infant death syndrome, respiratory infections, diabetes, obesity, cardiovascular disease, and cancer, and improve cognitive performance [2,3]. Remarkable new findings mainly attribute these beneficial attributes to milk’s bioactive components, mainly referring to the multifunctional macromolecules: lacto globulin, immunoglobulin, lactoferrin, lysozyme [4], and the novel promising extracellular vesicle (EV) [5,6]. Milk EVs have been recommended as relatively stable bioactive food compounds in recent research [7].

EVs are heterogeneous in size and source, including inward budding exosomes and outward budding microvesicles, as well as apoptotic bodies [8]. EVs naturally exist in various bio fluids, such as blood, saliva, urine, amniotic fluid, and milk, and facilitates their regulation of different physiological and pathological intercellular communications [9]. The regulating mechanisms underlying these processes may be enacted through the wrapped proteins (e.g., cytoskeletal, cytosolic, and plasma membrane proteins), lipids (e.g., sphingomyelin, phosphatidylserine, cholesterol and glycosphingolipids), and nucleotides, especially miRNAs [10,11]. Specific miRNAs exert significant influence on mammalian gene expression and protein synthesis as key regulatory factors, involving in cell cycling, proliferation, and differentiation, programmed cell death, immune activating and sensing nutrient stress [12,13,14].

New prevailing techniques such as genomics, metabolomics, proteomics make it possible to clarify the milk components and further illustrate the symbiotic relationship between the mother and the infant [2]. The grow of “nutrigenomics”, elucidating the metabolism of nutrients by high-throughput sequencing technologies, proved another vital role of milk besides providing nutrients, namely intervening in genetics [15]. A previous study supported the role of milk as an epigenetic “doping system” during mammalian development [16]. It is widely accepted that although cow milk is generally more often consumed by humans, sheep milk is more suitable for infants, partially because of the human-like protein and fat content in sheep milk [4]. However, compared with cow milk, few studies have focused on the sheep milk EVs, which wrap selected types of functional proteins and fat, particularly abundant regulatory miRNAs. This study clarified the sheep milk EV-miRNA profiling by sequencing and made a comparison with our previous research on cow milk (GSE142145, https://www.ncbi.nlm.nih.gov/geo/query/acc.cgi?acc=GSE142145). The results of this study provide more documents for the milk bioactive components in different species.

## 2. Materials and Methods

### 2.1. Ethics Statement

This research was approved by the State Key Laboratory of Animal Nutrition, Institute of Animal Science, Chinese Academy of Agriculture Sciences, Beijing, China. All the operations were strictly according to the Directions for Caring of Experimental Animals from the Ministry of Science and Technology, China ([2006] no. 398).

### 2.2. Milk Collection and Whey Preparation

Milk samples of 3 extensive farming sheep (Erdos breed; 2-year-old; 2 parity; mid-lactation) were collected from the Inner Mongolia Academy of Agriculture and Animal Husbandry Sciences. The milk mixture at 08:00 and 20:00 of each sheep was equally mixed together as a sample. All the samples were centrifuged at 2000× *g* for 10 min at room temperature to remove proteins, cells and debris. The translucent middle layer was moved to a new tube and centrifuged at 10,000× *g* for 30 min at room temperature to remove proteins and debris. Notably, the upper layer and lower pellet contained contaminating material that could compromise the quality of EVs. Thus, disturbance should be minimized. The middle layer of milk was centrifuged twice at 10,000× *g* for 10 min respectively at room temperature to further remove proteins and get milk whey.

### 2.3. Isolation of EVs from Milk

The Total Exosome Isolation Kit (from other body fluids) (Invitrogen, catalog no. 4484453, Waltham, MA, USA) was used to isolate sheep milk EVs. All the operations were strictly conducted in accordance with the instructions. Briefly, 400 μL milk whey was taken as a response system and added with 400 μL PBS to clarify milk sample. The clarified milk was added 400 μL Reagent and pipetting up and down until the solution was homogenous. After 30 min of incubation at room temperature, the mixture was centrifuged at 10,000× *g* for 10 min. The supernatant was discarded by pipette. EVs were settled on the bottom of the tube. The EVs were resuspended with 50 μL phosphate buffered solution and centrifuged at 10,000× *g* for 5 min. The EVs were dissolved in the supernatant and transferred in a new tube for further study.

### 2.4. Transmission Electron Microscopy

The milk EV pools from 3 sheep were chosen to carry out the test. The EVs were resuspended with 2% paraformaldehyde (catalog no. 158127, Sigma, St. Louis, MO, USA) instead of phosphate buffered solution, and 5 μL EVs were dropped onto the copper wire, standing at room temperature for 20 min. After being rinsed with phosphate buffered solution 3 times, the EVs were fixed with 1% glutaraldehyde solution (catalog no. G6257, Sigma, St. Louis, MO, USA) for 5 min. Then, the EVs were rinsed 10 times with distilled water and negative dyed with 5.4% uranyl acetate (catalog no. 22400, Electron microscopy sciences, Hatfield, PA, USA) for 5 min. After drying, it was observed using a Transmission Electron Microscope (TEM) (catalog no. FEI Tecnai G2 F20 S-TWIN, Hillsborough, OR, USA).

### 2.5. Particle Size Distribution

Isolated EVs were analyzed by Zeta View Electrophoresis and Brownian Motion Video Analysis Laser Scattering Microscopy (catalog no. S/N 17-310, Particle Metrix, Germany) to identify their physical characterization. The EVs were diluted with phosphate buffer saline and the dilution factor was 20,000. The operations were carried out strictly according to the operating procedures. The software was ZetaView 8.04.02, with camera 0.743 μm/px.

### 2.6. Small RNA Library Construction and Illumina Hiseq Sequencing

Milk RNA was extracted using the miRNeasy Mini Kit (catalog no. 217004, QIAGEN, Germany) following the manufacturer’s instructions. TruSeq Small RNA Sample Prep Kits (catalog no. RS-200-0012, Illumina, San Diego, CA, USA) were used to generate small RNA libraries. Briefly, 3′ adapter and 5′ adapter were ligated to the end of the RNA successively. Reverse transcription and amplification were performed with a polymerase chain reaction machine (catalog no. MyCycler, BIO-RAD, Berkeley, CA, USA). DNA loading dye (catalog no.LC6678, Invitrogen, Waltham, MA, USA) was used to purify the cDNA constructs by RNA gel electrophoresis. The purified cDNA constructs between 147 and 157 nt were recovered with Pellet Paint NF Co-Precipitant (catalog no. 70748, Merck Millipore, Beerlika, MA, USA). The size and purity of the libraries were checked by Agilent Technologies 2100 Bioanalyzer 2100 (catalog no. G2939A, Agilent, Santa Clara, CA, USA). The small RNA libraries were sequenced by Illumina Hiseq Sequencing at Shanghai Ouyi Biomedical Technology co., LTD (Shanghai, China).

### 2.7. Bioinformatics Analysis

The process of bioinformatics analysis was conducted as we did before [17]. Brifely, the low-quality reads, 5′ primer contaminants and poly (A) were removed from raw reads by Cutadapt (version 1.14) (https://cutadapt.readthedocs.io/en/stable/) and the sequences between 15 and 41 nt were retained. FASTX Toolkit (version 0.0.13) (http://hannonlab.cshl.edu/fastx_toolkit/) and NGS QC Toolkit (version 2.3.2) (https://omictools.com/ngs-qc-toolkit-tool) were used to control the quality of Q20 and filter the reads with N bases. Then high-quality clean reads were obtained and used for subsequent analysis. The clean reads were aligned to GenBank databases (http://www.ncbi.nlm.nih.gov/genbank/) to match the reads with reference genome of cow (*Bos taurus*, RefSeq assembly: GCF_002263795.1) and sheep (*Ovis aries*, RefSeq assembly: GCF_002742125.1) respectively, and the percentages of reads aligned to the genome were calculated. The clean reads were further compared with Rfam (version.10.0) (http://www.sanger.ac.uk/software/Rfam) to annotate and filter all types of small RNA. BLAST (https://blast.ncbi.nlm.nih.gov/Blast.cgi) and Rfam (version.10.0) were used to remove transcripts and repeat sequences. The known miRNAs and their structures were annotated by miRBase (version.22.0) database (http://www.mirbase.org/). The unannotated sequences were matched with miRDeep2 (https://www.mdc-berlin.de/content/mirdeep2-documentation) to identify novel miRNAs and their hairpin-structures were predicted by RNAfold (http://rna.tbi.univie.ac.at/cgi-bin/RNAWebSuite/RNAfold.cgi). Bioinformatics and Evolutionary Genomics (http://bioinformatics.psb.ugent.be/webtools/Venn/) was used to calculate the intersections of the miRNA types and draw Venn diagrams. The target genes of the top 20 miRNAs in cow and sheep were predicted by miRanda software (http://miranda.org.uk). Kyoto Encyclopedia of Genes and Genomes (KEGG) pathway analyses (https://www.kegg.jp) were performed with predicted target genes and the significance of enrichments in pathways were calculated by hypergeometric distribution test.

### 2.8. Polymerase Chain Reaction (PCR) Verification

We randomly selected 8 known miRNAs to verify the accuracy of the sequencing results by real-time quantitative PCR: oar-miR-199a-3p, oar-let-7c, oar-let-7i, oar-miR-143, oar-miR-200b, oar-miR-10a, oar-miR-103, and oar-let-7f. The primers of the selected miRNAs were presented in Table 1. The cel-miR-39-5p was used as an external standard. The first strand cDNA was synthesized by RevertAid First Strand cDNA Synthesis Kit (catalog no. K1622, Thermo Scientific, Waltham, MA, USA). PCR-iQ5 system (Bio-Rad, Berkeley, CA, USA) was used to perform the reaction. The mixture of 2 μL cDNA (25 ng), 1 μL specific forward primer, 1 μL universal primer, 12.5 μL SYBR Premix Ex TaqTM II (TaKaRa, Dalian, China) and 8.5 μL water was in a reaction system. Each miRNA had three technical replicates. The relative expression of miRNA was calculated based on a standard curve using the 2^−ΔΔCT^ method [18].

## 3. Results

### 3.1. Micrograph and Particle Size Distribution of Sheep Milk EVs

**Figure 1:**
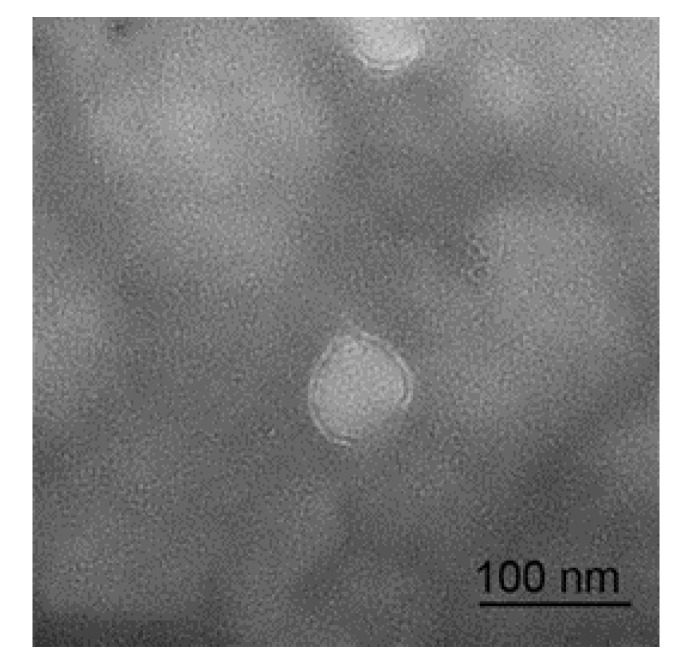
Micrograph of sheep milk extracellular vesicles (EVs).

**Figure 2:**
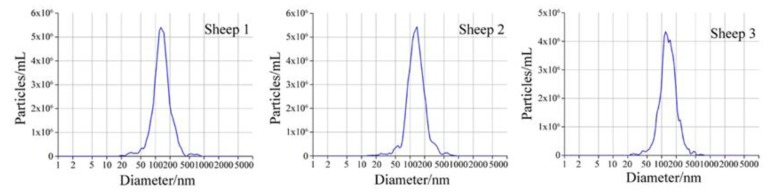
Nanoparticle tracking analysis of sheep milk extracellular vesicles (EVs).

The micrograph of a sheep milk EV is shown in Figure 1. According to the micrograph, the diameter of a sheep milk EVs is about 50 nm and rounded in shape. Nanoparticle tracking analysis of sheep milk EVs is shown in Figure 2. The number of traced particles by the video operator was 1143 ± 50.3 and the average counted particles per frame was 133 ± 0.9. The sheep milk EV diameter was peaked in 131.2 ± 0.84 nm. The concentration of sheep milk EVs was 1.3 ± 0.09 × 10^12^ particles/mL.

### 3.2. Small RNA-Loading EVs Were Abundant in Sheep Milk

**Figure 3:**
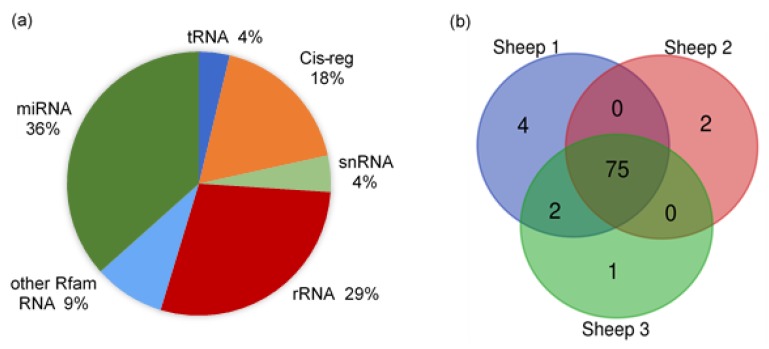
The proportion of small RNAs and annotated types of miRNAs in sheep milk EVs. (**a**) The proportion of small RNAs in sheep milk EVs. (**b**) The annotated types of miRNAs in sheep milk EVs.

The raw reads of all samples were from 18.39 to 30.90 M, with clean reads counted between 16.20 and 9.99 M. The genome alignment rates of all samples were distributed from 66.85% to 89.08%, and the miRNA alignment rates were between 4.24% and 10.50%. The proportion of small RNAs and annotated types of miRNAs in sheep milk EVs are shown in Figure 3. There were several types of small RNAs in the sheep milk EVs, including miRNA, tRNA, Cis-regulatory elements (Cis-reg), rRNA, snRNA, and other Rfam RNA. The miRNAs accounted for about 36% of the small RNAs, while rRNA occupied about 29%. The other Rfam RNAs, tRNAs, snRNAs and Cis-regs took up about 35% in total. The number of miRNA types in each sample annotated by *Ovis aries* database is listed in Figure 3b. A total of 84 types of known miRNAs, with 601 novel miRNAs, were identified in sheep milk EVs, with 75 shared types of miRNAs in all samples.

### 3.3. The Top 20 Highly Expressed EVs in Cow Milk and Sheep Milk EVs

The top 20 miRNAs with the highest expression in cow and sheep milk EVs are shown in Table 2. Among all the top 20 EV-miRNAs were miR-26a, miR-191, let-7f, let-7b and miR-10b, which were highly expressed in both cow and sheep milk. Moreover, 13 bovine miRNAs in the top 20, accounting for about 76% of the totality, were immune-related: bta-miR-26a, bta-miR-191, bta-miR-423-5p, bta-let-7f, bta-miR-30d, bta-let-7a-5p, bta-miR-27b, bta-let-7b, bta-miR-92a, bta-miR-125a, bta-miR-451, bta-miR-150, and bta-miR-21-5p. Meanwhile, 14 sheep miRNAs in the top 20, accounting for about 98%, were immune-related: oar-miR-148a, oar-let-7b, oar-let-7a, oar-miR-21, oar-let-7c, oar-let-7i, oar-miR-26a, oar-let-7f, oar-miR-125b, oar-miR-143, oar-miR-27a, oar-miR-181a, oar-miR-191, and oar-miR-200b. Besides let-7f and let-7b, sheep milk EVs contained more let-7 family miRNAs, including oar-let-7a, oar-let-7c, oar-let-7i and oar-let-7g. Meanwhile, the highly expressed oar-miR-125b and oar-miR-10b in sheep milk EVs belonged to miR-10 family.

### 3.4. Target Gene Function Analysis of the Top 20 EV-miRNAs in Cow and Sheep Milk

**Figure 4:**
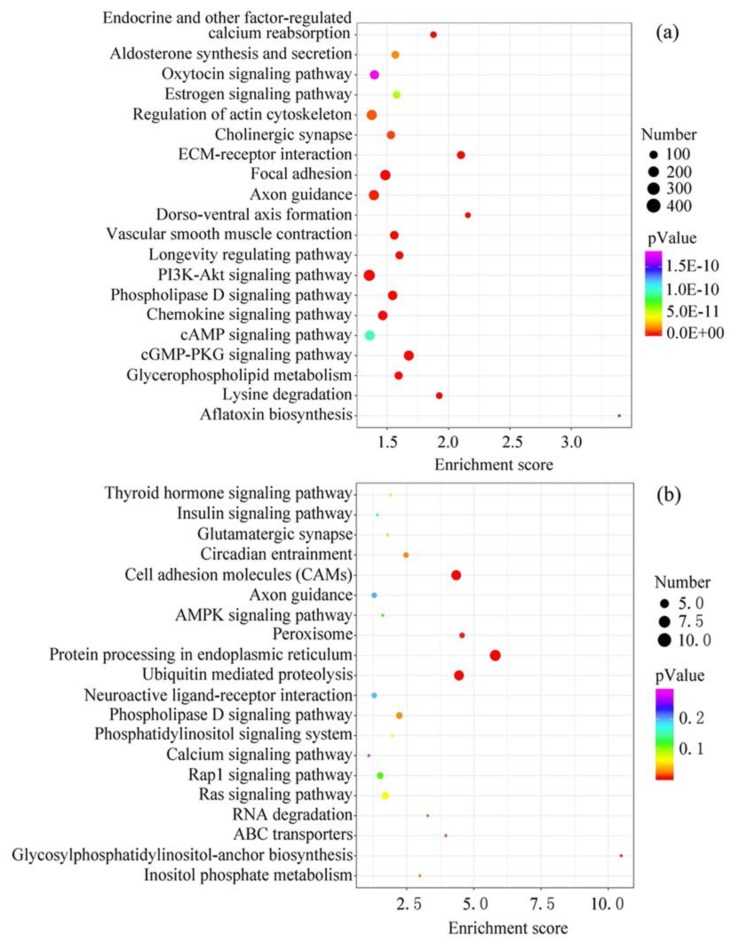
KEGG pathway analysis of the target genes of the top 20 EV-miRNAs. (**a**) Cow milk. (**b**) Sheep milk.

The target genes of the top 20 EV-miRNAs in cow and sheep milk were predicted and conducted function analysis by KEGG (Figure 4). The number of target genes with significant changes in KEGG pathway was 6213 in cow milk EVs, and only 88 in sheep milk EVs. In addition, the total number of genes in the KEGG pathway was 21,096 in cow milk EVs, and 14,757 in sheep milk EVs, which also resulted in the remarkable difference in the *p*-Value in different ruminants. The top 20 pathways in cow milk were related to aflatoxin biosynthesis, ECM-receptor interaction, cGMP-PKG signaling pathway, focal adhesion, phospholipase D signaling pathway, lysine degradation, vascular smooth muscle contraction, dorso-ventral axis formation, chemokine signaling pathway, glycerophospholipid metabolism, PI3K-Akt signaling pathway, longevity regulating pathway, endocrine and other factor-regulated calcium reabsorption, axon guidance, cholinergic synapse, regulation of actin cytoskeleton, aldosterone synthesis and secretion, estrogen signaling pathway, cAMP signaling pathway and oxytocin signaling pathway. Meanwhile, the top 20 pathways in sheep milk were protein processing in endoplasmic reticulum, ubiquitin mediated proteolysis, cell adhesion molecules, glycosylphosphatidylinositol-anchor biosynthesis, peroxisome, ABC transporters, RNA degradation, inositol phosphate metabolism, circadian entrainment, phospholipase D signaling pathway, Ras signaling pathway, phosphatidylinositol signaling system, thyroid hormone signaling pathway, glutamatergic synapse, Rap1 signaling pathway, AMPK signaling pathway, insulin signaling pathway, neuroactive ligand-receptor interaction, axon guidance and calcium signaling pathway. Although KEGG analysis showed different potential functions in cow and sheep milk EV-miRNAs, there were still some shared pathways among different species—lipid metabolism (phospholipase D, glycerophospholipid and glycosylphosphatidylinositol), calcium metabolism, and nerve conduction (axon guidance and synapse).

### 3.5. Validation of Selected miRNAs by Quantitative RT-PCR

**Figure 5:**
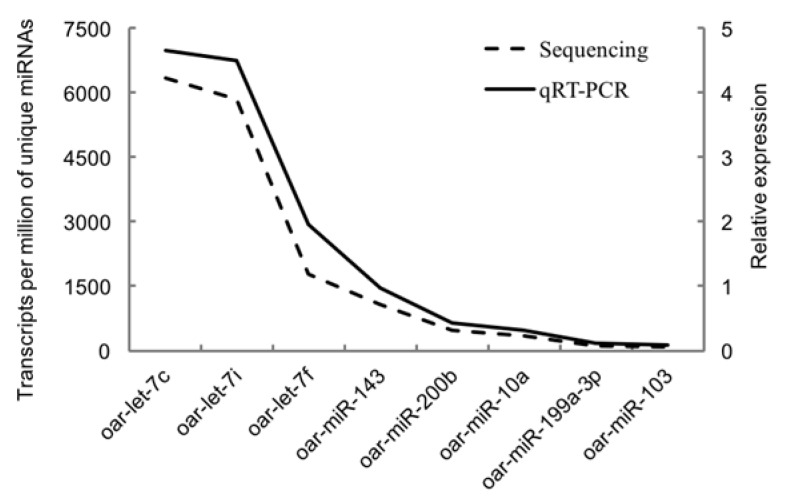
Validation results of the selected miRNAs by quantitative RT-PCR.

Figure 5 showed the validation results of the selected miRNAs by quantitative RT-PCR. The relative expression of all eight miRNAs (oar-miR-199a-3p, oar-let-7c, oar-let-7i, oar-miR-143, oar-miR-200b, oar-miR-10a, oar-miR-103, and oar-let-7f) was positively correlated with the reads of their miRNAs generated from sequencing. The similar trends of quantitative PCR results and miRNA-sequencing results indicated the accuracy of the sequencing data.

## 4. Discussion

Milk composition is influenced by many factors, such as species and genetics, lactation stages, environmental conditions, and nutritional status [4]. Emerging genomics tools, especially next generation sequencing, facilitated the identification of pathways through which the milk components regulated maternal mammary gland development and infant growth, laying the foundation of nutrigenomics [24]. The outputs of ruminant nutrigenomics could benefit the performance of dairy cattle and modify the milk components for the increasing demands of “healthy” food of consumers [25]. Sheep milk was more suitable for infants than cow milk because it contained higher and human-like contents of minerals, vitamins, non-protein nitrogen, and essential bioactive proteins include immunoglobulins, transferrin, lactoferrin, ferritin, proteose peptones, prolactin, calmodulin, and folate-binding proteins [26]. However, few studies have focused on the novel sheep milk bioactive EVs. Therefore, this study clarified the sheep milk EV-miRNA profiling by next generation sequencing and made a comparison with our previous research on that of cow milk (GSE142145), providing references for sheep milk active compounds and nutrigenomics. We found 84 *Ovis aries* miRNAs in the sheep milk EVs by aligning with the 153 mature miRNAs in the miRBase database, with 601 novel miRNAs. Accordingly, alignment with the 1025 mature miRNAs in the miRBase database, 276 *Bos taurus* miRNAs were identified in cow milk EVs by RNA sequencing, with 503 novel miRNAs (GSE142145). In addition, Chen et al. found there were 176 *Sus scrofa* miRNAs, with 315 novel miRNAs, in the pig milk EVs aligned with a total of 457 miRNAs in miRBase database [27]. Similarly, Zhou et al. found 639 *Homo sapiens* miRNAs in the human milk EVs aligned with a total of 2654 miRNAs in the database [28]. Since research on milk EV-miRNAs among species is limited, and is especially scarce in sheep, we could not evaluate what the exact numbers were in the milk EV-miRNAs of specific species. At the same time, before we could figure that out, it was also important to expand the database we used.

Milk is an abundant source of evolutionary conserved EV-miRNAs between mammals [29]. Therefore, function studies from other species on the highly conserved miRNAs might also be applied to cow or sheep. We found that miR-26a, miR-191, let-7f, let-7b and miR-10b were highly expressed in both cow and sheep milk EVs, implying their possible significance in neonatal growth and development. In addition, compared with cow milk EVs, those of sheep contained more let-7 family and miR-10 family miRNAs. The miR-26a could target the transcription factor KLF4 and regulate innate immune signaling [30]. It was reported that bovine miR-191 might target genes that accelerate the developments of gut tissue and immune system [31]. As the first known human miRNA, let-7 and its family members were highly conserved across species, both in sequences and functions involving mammalian cell proliferation and differentiation, brain and limb development, and temporal regulation [32]. The miR-10 family was also highly conserved and evolutionarily ancient, playing an essential role in global protein synthesis by enhancing the translation of 5′-terminal oligo pyrimidine mRNAs [33]. In summary, these miRNAs across species might be closely related to the maintenance of life, such as cell proliferation and differentiation, protein synthesis, and the development of the gastrointestinal tract and immune system.

The most highly expressed miRNAs in the milk EVs were well-characterized and immune-related in this study, both in bovines and sheep, proving the immune function of milk in the nucleic acid components. The top expressed miR-148a in sheep was a critical regulator of B cell tolerance and systemic autoimmunity [34]. Let-7b could target Toll-like receptors (TLR) and regulate the activation of NF-*κ*B and its downstream genes related to inflammation and immune responses during infection [35]. The miR-21 in sheep milk EVs could activate the survival of memory T-cells and downregulate CC-chemokine receptor 7 protein expression in naive T-cells, thus divergently regulating the T-cell biology [36]. Similarly, miR-191 could also promote CD4+ regulatory T cell survival following common γ chain signaling [37]. In addition, the highly expressed oar-miR-125b was reported as an essential regulator for normal B-cell development, and the miR-27a in sheep milk EVs could mediate innate immune responses by negatively regulating IL-10 expression and enhancing the expression of pro-inflammatory cytokines in TLR 2 and TLR 4 [38].

The pathway analysis of the target genes of the highly expressed sheep milk EV-miRNAs mainly referred to protein processing in endoplasmic reticulum and ubiquitin-mediated proteolysis, indicating that the non-coding RNAs might regulate the protein metabolism of lambs. Moreover, cell adhesion molecules also showed dramatic significance in all the sheep EV-miRNA target pathways, which were expressed on the cell surface and facilitated the interaction and the capture of EVs, verifying the EV source of sheep milk [39]. Phospholipase D signaling pathway also held an important position in all the targets of sheep EV-miRNAs. In fact, EVs contained many types of active lipolytic moieties, especially phospholipases, to form bioactive lipid mediators [40,41]. Specifically, phospholipase D2 was abundant in EVs and its activity correlated with the amount of EVs [41], and the phospholipase D signaling pathway was also present in the cow milk, implying the shared nature of milk EVs among species. Furthermore, the shared phospholipase D and glycerophospholipid metabolism in cow milk EVs, and glycosylphosphatidylinositol-anchor biosynthesis in sheep milk EVs all referred to lipid metabolism, which is essential to the structure of EV membranes [10]. Interestingly, other shared pathways of sheep and cow included axon guidance and synapse, suggesting that milk EVs might be related to the neonatal transmission of neural signals.

It is worth noting that processing and storage could affect the stability of milk EV-miRNAs, including pasteurization, homogenization and heating in the microwave [42]. Since cheese-making is a complicated fermentation process, the changes in sheep milk EV-miRNA bioavailability in this process need further study. In addition, although most people agreed on the beneficial effects of cow milk EVs, recent researchers found that pasteurized ruminant milk EVs might introduce new pathogens to human by the miRNA cargos [43], which emphasized the importance of further research on milk EVs.

## Figures and Tables

**Table 1:** Primers used to detect miRNA expression by qRT-PCR.

miRNA Name	Primer Sequences (5′-3′)	Length (nt)
celmiR-39-5p	AGCTGATTTCGTCTTGGTAATA	22
oar-miR-199a-3p	CGACAGTAGTCTGCACATTGGTTAA	25
oar-let-7c	CACGGCTGAGGTAGTAGGTTGTATG	25
oar-let-7i	CCGTGAGGTAGTAGTTTGTGCTGTT	25
oar-miR-143	ACGGTGAGATGAAGCACTGTAGC	23
oar-miR-200b	TAATACTGCCTGGTAATGATG	21
oar-miR-10a	TACCCTGTAGATCCGAATTTG	21
oar-miR-103	AGCAGCATTGTACAGGGCTATG	22
oar-let-7f	TGAGGTAGTAGATTGTATAGTT	22

The primer sequence of cel-miR-39-5p referred to Gu et al. [19]. The primer sequence of oar-miR-199a-3p, oar-let-7c, oar-let-7i, and oar-miR-143 referred to Jin et al. [20]. The primer sequence of oar-miR-200b and oar-miR-10a referred to Hou et al. [21]. The primer sequence of oar-miR-103 referred to Wu et al. [22]. The primer sequence of oar-let-7f referred to Du et al. [23].

**Table 2:** The top 20 highly expressed miRNAs in cow and sheep milk EVs.

Number	Cow	Sheep
1	bta-miR-26a	oar-miR-148a
2	bta-miR-191	oar-let-7b
3	bta-miR-486	oar-let-7a
4	bta-miR-151-5p	oar-miR-21
5	bta-miR-423-5p	oar-let-7c
6	bta-let-7f	oar-let-7i
7	bta-miR-30d	oar-miR-26a
8	bta-let-7a-5p	oar-let-7f
9	bta-miR-27b	oar-miR-125b
10	bta-miR-22-3p	oar-miR-143
11	bta-let-7b	oar-miR-30a-5p
12	bta-miR-99a-5p	oar-miR-27a
13	bta-miR-92a	oar-miR-127
14	bta-miR-125a	oar-miR-181a
15	bta-miR-451	oar-let-7g
16	bta-miR-150	oar-miR-191
17	bta-miR-10b	oar-miR-200c
18	bta-miR-21-5p	oar-miR-30a-3p
19	bta-miR-30e-5p	oar-miR-200b
20	bta-miR-3600	oar-miR-10b

## Abbreviations

miRNA	microRNA
EV	Extracellular Vesicle
KEGG	Kyoto Encyclopedia of Genes and Genomes
PCR	Polymerase Chain Reaction
AMPK	Adenosine Monophosphate Activated Protein Kinase
TLR	Toll-like receptors
